# Half-Space Sound Field Reconstruction Based on the Combination of the Helmholtz Equation Least-Squares Method and Equivalent Source Method

**DOI:** 10.3390/s24144651

**Published:** 2024-07-17

**Authors:** Laixu Jiang, Yingqi Xi, Yingying Hu, Guo Wang, Jingqiao Liu

**Affiliations:** Marine Design and Research Institute of China, Shanghai 200011, China; findme_305@sohu.com (Y.X.); h165607@163.com (Y.H.); wg198885@126.com (G.W.); 135016304322@139.com (J.L.)

**Keywords:** half-space sound field, sound field reconstruction, surface impedance, near-field acoustic holography, Helmholtz equation least squares method, equivalent source theory

## Abstract

In practical conditions, near-field acoustic holography (NAH) requires the measurement environment to be a free sound field. If vibrating objects are located above the reflective ground, the sound field becomes non-free in the presence of a reflecting surface, and conventional NAH may not identify the sound source. In this work, two types of half-space NAH techniques based on the Helmholtz equation least-squares (HELS) method are developed to reconstruct the sound field above a reflecting plane. The techniques are devised by introducing the concept of equivalent source in HELS-method-based NAH. Two equivalent sources are tested. In one technique, spherical waves are used as the equivalent source, and the sound reflected from the reflecting surface is regarded as a linear superposition of orthogonal spherical wave functions of different orders located below the reflecting surface. In the other technique, some monopoles are considered equivalent sources, and the reflected sound is considered a series of sounds generated by simple sources distributed under the reflecting surface. The sound field is reconstructed by matching the pressure measured on the holographic surface with the orthogonal spherical wave source in the vibrating object and replacing the reflected sound with an equivalent source. Therefore, neither technique is related to the surface impedance of the reflected plane. Compared with the HELS method, both methods show higher reconstruction accuracy for a half-space sound field and are expected to broaden the application range of HELS-method-based NAH techniques.

## 1. Introduction

The Helmholtz equation least squares method (HELS) as a method of near-field acoustic holography (NAH) has become an effective tool for sound source identification and sound field reconstruction due to the simple formula, efficient calculation, and adaptable applications [1,2]. In the HELS method, the sound field is approximated based on a combination of basis functions in orthogonal spherical waves, and the number of basis functions is determined with the least square method. However, the NAH technique is primarily developed for reconstructing the free sound field [3,4,5,6,7,8]. A free sound field is an external sound field with no interference source and no boundary, or an internal sound field with no disturbance source is closed. In practice, most vibrating sources are located over the ground, and the sound field is a bounded half-space sound field. The boundary may be rigid or show strong sound-absorption characteristics, and therefore, the effect of reflection at the boundary should be considered in reconstructing a half-space sound field. In this case, the only free field HELS techniques cannot properly reconstruct this half-space sound field, which greatly limits applying the HELS-NAH technique in situ.

There are two reconstruction methods: the sound field separation method and the rigid half-space Green’s function method [9,10]. The former method is adopted to eliminate the influence of the reflecting surface in the half-space sound field, and the position and impedance characteristics of the reflecting surface are not required; however, a measurement plane is required, and it must be a closed surface enclosing the vibrating object. Moreover, either the double-sided measurement of the sound pressure [11] or vibration velocity [12] or the single-sided measurement of both of these parameters is necessary [13]. While the rigid half-space Green’s function method can effectively reconstruct the half-space sound field [14,15], it requires predictions of the position and impedance characteristics of the reflecting surface.

In this work, to broaden the application of the F-HELS method in practical engineering (non-free field acoustic field reconstruction), two half-space NAH techniques based on the HELS method are developed to reconstruct the acoustic quantity (pressure or velocity) in the sound field over a reflecting surface. Both techniques are developed by introducing the concept of equivalent sources in the HELS method. In one technique, spherical waves are used as the equivalent source, and the sound reflected from the reflecting surface is regarded as a linear superposition of orthogonal spherical wave functions of different orders located below the reflecting surface. Therefore, the sound directly radiated by the vibrating object and reflected sound from the reflecting surface can be expressed as linear superpositions of orthogonal spherical wave functions. Hereafter, this method is called the H-HELS method for simplicity. In the other technique, some monopoles are used as equivalent sources, and the reflected sound is regarded as a series of simple source radiations distributed under the reflecting surface. Therefore, the directly radiated sound can be expressed as a linear superposition of a series of orthogonal spherical wave functions, and the reflected sound can be expressed as a linear superposition of a series of simple sources. Hereafter, this method is called the E-HELS method for simplicity. Applying these two technologies does not require a priori knowledge about the reflected surface impedance.

## 2. Theories of H-HELS and E-HELS

### 2.1. Basic Theory of Free-HELS Method

The basic concept of the F-HELS method is that the sound field radiated by a vibrating source can be represented by a series of linear combinations of orthogonal spherical wave functions in the spherical coordinate system, and the coefficients are solved using the least-squares method by matching measuring pressures. The sound pressure and normal velocity in a free field can be expressed as

(1) $$P_{free}\left(x\right)=\rho c\sum_{j=1}^{J}\Psi_{pj,free}\left(x\right)C_{j},$$

(2) $$V_{free}\left(x\right)=\sum_{j=1}^{J}\Psi_{vj,free}\left(x\right)C_{j},$$

where ρ is the medium density, *c* is the speed of sound, $\Psi_{pj,free}(x)\;\mathrm{and}\;\Psi_{vj,free}(x)$ are basis function special solutions of the Helmholtz equation, $C_{j}$ is the corresponding coefficient, and J is the number of expansion terms in the basis function. In the spherical coordinate system, $\Psi_{pj,free}(x)$ and $\Psi_{vj,free}(x)$ are spherical wave functions, which can be expressed as

(3) $$\Psi_{pj,free}\left(x\right)=\Psi_{n,m}\left(\gamma ,\theta ,\varphi \right)=h_{n}^{\left(1\right)}\left(kr\right)\sqrt{\frac{\left(2n+1\right)\left(n-m\right)!}{4\pi \left(n+m\right)!}}P_{n,m}(cos\theta )\left\{\begin{array}{c} \cos\left(m\varphi \right)\\ \sin\left(m\varphi \right) \end{array}\right.,$$

(4) $$\Psi_{vj,free}\left(x\right)=i\frac{\partial h_{n}^{\left(1\right)}\left(kr\right)}{\partial \left(kr\right)}\sqrt{\frac{\left(2n+1\right)\left(n-m\right)!}{4\pi \left(n+m\right)!}}P_{n,m}(cos\theta )\left\{\begin{array}{c} \cos\left(m\varphi \right)\\ \sin\left(m\varphi \right) \end{array}\right.,$$

(5) $$n=floor\left(\sqrt{j}\right)l=j-n^{2},$$

(6) $$m=\left\{\begin{array}{c} l/2\;\;\;\;\;\;\;\;\;\;\;\;\;l=0,2,4\\ \left(l-1\right)/2\;\;\;\;\;\;\;\;\;l=1,3,5\;\;\;\;\;\;\; \end{array}\right.,$$

where i is the imaginary unit number ($i=\sqrt{-1}$), $h_{n}^{\left(1\right)}$ is the first Hankel function of the *n*th order, $P_{n,m}\left(\cos\theta \right)$ is the associated Legendre function, and k=ω∕c is the wavenumber, where ω is the angular frequency.

According to the superposition principle of orthogonal spherical waves, if there are M measuring points on hologram plane *H* in the free field, Equations (1) and (2) can be expressed in matrix forms:

(7) $$P_{free,H}=\rho c\Psi_{p,free,H}C_{H},$$

(8) $$V_{free,H}=\Psi_{v,free,H}C_{H},$$

where

$$\Psi_{p,free,H}={\left[\Psi_{p,free,H}^{\left(1\right)}\ldots \cdot \Psi_{p,free,H}^{\left(J\right)}\right]}_{M\times J},$$

$$\Psi_{v,free,H}={\left[\Psi_{v,free,H}^{\left(1\right)}\ldots \cdot \Psi_{v,free,H}^{\left(J\right)}\right]}_{M\times J},$$

$$C_{H}={\left[{\left(C^{\left(1\right)},C^{\left(2\right)},\cdots C^{\left(J\right)}\right)}^{T}\right]}_{J\times 1}.$$

The least-squares solution of Equation (7) is

(9) $${\left\{C_{H}\right\}}_{J\times 1}={\left[{\left(\Psi_{p,free,H}\right)}_{J\times M}^{H}{\left(\Psi_{p,free,H}\right)}_{M\times J}+\varepsilon E\right]}^{-1}{\left(\Psi_{p,free,H}\right)}_{J\times M}^{H}{\left\{P_{free,H}\right\}}_{M\times 1},$$

where $\Psi_{p,free,H}$ and $\Psi_{v,H,free}$ are the basis function matrix of pressure and velocity on the measurement plane. The superscript ‘H’ denotes Hermitian transposition, ε is a regularization parameter, *E* is a unit matrix, and the exponent ‘−1’ indicates the inverse matrix.

The sound pressure and the normal velocity of the vibrating object surface and any external field point $X_{R}\left(R=1,2,3\cdots M_{R}\right)$ can be reconstructed using Equations (7) and (8) when the coefficient vector $C_{H}$ is solved, shown as follows:

(10) $$P_{free,R}=\Psi_{p,free,R}{\left[{\left(\Psi_{p,free,H}\right)}_{J\times M}^{H}{\left(\Psi_{p,free,H}\right)}_{M\times J}+\varepsilon E\right]}^{-1}{\left(\Psi_{p,free,H}\right)}_{J\times M}^{H}{\left\{P_{free,H}\right\}}_{M\times 1},$$

(11) $$V_{free,R}=\Psi_{v,free,R}{\left[{\left(\Psi_{p,free,H}\right)}_{J\times M}^{H}{\left(\Psi_{p,free,H}\right)}_{M\times J}+\varepsilon E\right]}^{-1}{\left(\Psi_{p,free,H}\right)}_{J\times M}^{H}{\left\{P_{free,H}\right\}}_{M\times 1},$$

where $\Psi_{p,free,R}\;\mathrm{and}\;\Psi_{v,free,R}$ are the pressure and normal velocity basis function matrix on the reconstruction plane in the free sound field, respectively.

### 2.2. Basic Theory of ESM

**Figure 1:**
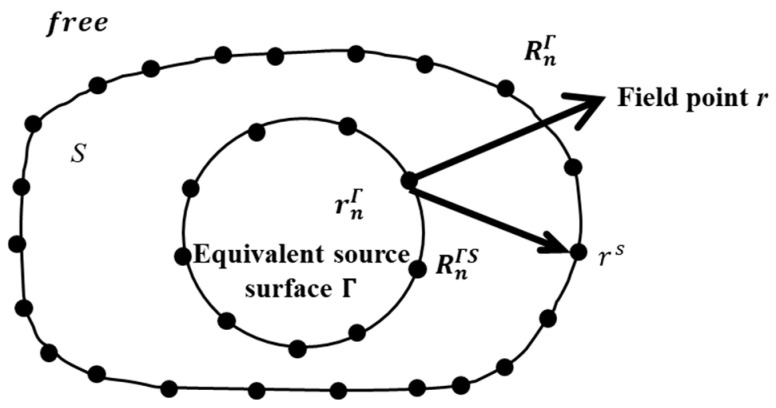
Schematic for the formulation of the free sound field by using the ESM.

The basic idea of ESM is to model the sound field by using a distribution of simple virtual sources [8]. When the vibrating object is in a free space field, shown in Figure 1, where the black dots in the inner circle indicate the monopole equivalent sources and the outer black dots indicate the points on the surface of the vibrating object. The sound pressure $P\left(r\right)$ at radiation field point r(x,y,z) and the normal velocity $V(r^{s})$ at a point on the surface $r^{s}(x^{s},y^{s},z^{s})$ of the vibrating object can be expressed as

(12) $$P(r)=i\rho \omega \sum_{n=1}^{N}g_{p,free}(r,r_{n}^{\Gamma })q_{n}^{\Gamma },$$

(13) $$V(r^{s})=\sum_{n=1}^{N}g_{v,free}(r^{s},r_{n}^{\Gamma })q_{n}^{\Gamma },$$

where N is the number of equivalent sources on the virtual surface $\Gamma ,\;q_{n}^{\Gamma }$ is the strength of the *n*th equivalent source on Γ, and $r_{n}^{\Gamma }=(x_{n}^{\Gamma },y_{n}^{\Gamma },z_{n}^{\Gamma })$ is the position of the *n*th equivalent source on Γ. Furthermore, $g_{p,free}(r,r_{n}^{\Gamma })$ and $g_{v,free}(r^{s},r_{n}^{\Gamma })$ are the pressure transfer function and the velocity transfer function, respectively, which connect the equivalent sources with the hologram pressure and the normal velocity on the surface of the vibrating object in the free sound field. They can be expressed as

(14) $$g_{p,free}(r,r_{n}^{\Gamma })=\frac{e^{ikR_{n}^{\Gamma }}}{4\pi R_{n}^{\Gamma }},$$

(15) $$G_{v,free}(r^{s},r_{n}^{\Gamma })=\frac{(1-ikR_{n}^{\Gamma S})e^{ikR_{n}^{\Gamma S}}}{4\pi {\left(R_{n}^{\Gamma S}\right)}^{2}}cos(\varphi_{n}^{\Gamma S}),$$

with

(16) $$cos(\varphi_{n}^{\Gamma S})=\frac{(r^{s}-r_{n}^{\Gamma })\cdot n^{S}}{\left|r^{s}-r_{n}^{\Gamma }\right|},$$

where ‘·’ denotes the dot product operator, $n^{S}$ is the normal unit vector on the surface of the vibrating object, and $i=\sqrt{-1}$. Furthermore, $R_{n}^{\Gamma }$ and $R_{n}^{\Gamma S}$ denote the distances from r and $r^{s}$ to $r_{n}^{\Gamma }$, respectively.

Under the assumption that there are M measuring points on the plane *H* in free space, one can rewrite Equation (12) in a matrix form as follows:

(17) $$P^{H}=i\rho \omega G_{p,free}^{\Gamma H}Q^{\Gamma },$$

where $G_{p,free}^{\Gamma H}$ is an *M × N* transfer matrix between the measuring pressure and the equivalent source, and $Q^{\Gamma }=\left[q_{1}^{\Gamma },\cdots ,q_{n}^{\Gamma },\cdots ,q_{N}^{\Gamma }\right]$ is the column vector of equivalent source strengths. After the regularization of Equation (17), it can be expressed as

(18) $$Q^{\Gamma }=\frac{1}{i\rho w}{\left[{\left(G_{p,free}^{\Gamma H}\right)}^{H}G_{p,free}^{\Gamma H}+\varepsilon E\right]}^{-1}{\left(G_{p,free}^{\Gamma H}\right)}^{H}P^{H},$$

Since obtaining the solution of equivalent source strength is an acoustic inverse problem, the measurement error is significantly amplified in the reconstruction process. Therefore, a regularization method is required to suppress the reconstruction error. After obtaining $Q^{\Gamma }$, the radiated pressure at any point $r^{f}(x^{f},y^{f},z^{f})$ in the free-space sound field and the normal velocity on the surface $r^{s}(x^{s},y^{s},z^{s})$ can be calculated as

(19) $$P^{f}={i\rho wG}_{p,free}^{\Gamma f}Q^{\Gamma },$$

(20) $$V^{S}=G_{v,free}^{\Gamma S}Q^{\Gamma },$$

where $G_{p,free}^{\Gamma f}$ is an $R^{f}\times N$ transfer matrix between the pressure at any point and the equivalent source on Γ, $G_{v,free}^{\Gamma S}$ is an $N^{S}\times N$ transfer matrix between surface normal velocities on S and the equivalent source on Γ, $N^{S}$ denotes the surface nodes on *S*, and $R^{f}$ is the number of reconstruction points for the pressure. The basic theory of the ESM described above is used in the E-HELS method to eliminate the sound reflected from the reflecting surface in a half-space field.

### 2.3. Basic Theory of H-HELS

**Figure 2:**
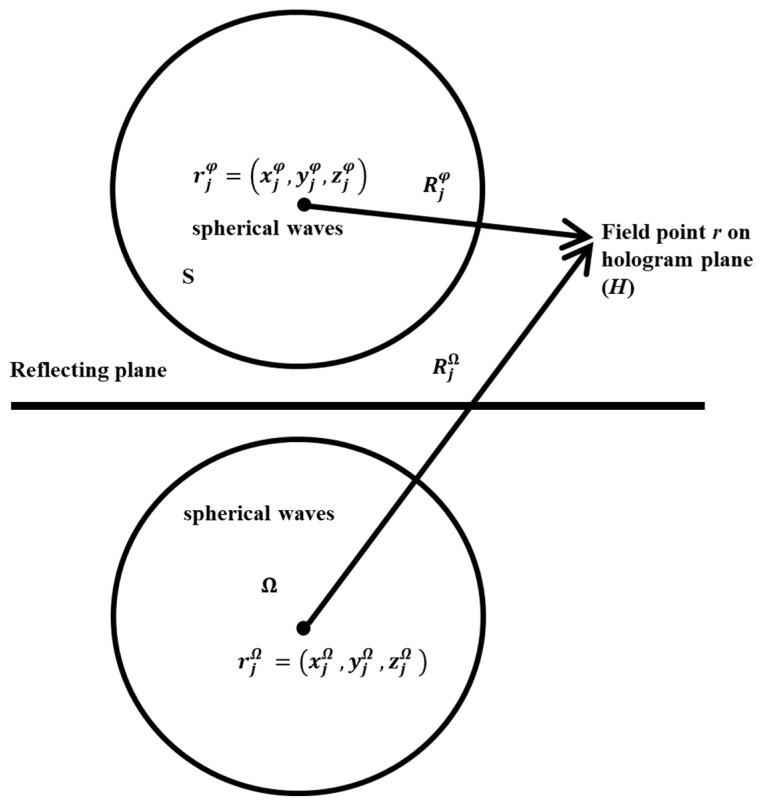
Diagram of H-HELS theory.

Previous studies have shown that introducing the half-space Green’s function into the traditional NAH can help to effectively reconstruct a half-space sound field. However, a precondition of this method is that the surface impedance of the reflective surface should be known. This section introduces the first technique proposed in this paper, H-HELS. In H-HELS, spherical waves are used as the equivalent source, and the sound reflected from the reflecting surface is regarded as a linear superposition of orthogonal spherical wave functions of different orders located below the reflecting surface. Then, the half-space sound field is reconstructed by matching the measuring pressure with an orthogonal spherical wave source in the vibrating object and an orthogonal spherical wave source located below the reflecting surface instead of the reflected sound. A schematic of the technique is shown in Figure 2.

In Figure 2, the pressure at field point r can be approximated as

(21) $$P\left(r\right)=\rho c\sum_{j=1}^{J}\Psi_{pj,free}\left({r,r}_{j}^{\varphi }\right)C_{j}^{\varphi }+\rho c\sum_{j=1}^{J}\Psi_{pi,free}\left({r,r}_{j}^{\Omega }\right)C_{j}^{\Omega },$$

where $C_{j}^{\varphi }$ and $C_{j}^{\Omega }$ are the coefficients of the basis functions, $r_{j}^{\varphi }=\left(x_{j}^{\varphi },y_{j}^{\varphi },z_{j}^{\varphi }\right)$ is the position of the orthogonal spherical wave source in the vibrating object, and $r_{j}^{\Omega }=\left(x_{j}^{\Omega },y_{j}^{\Omega },z_{j}^{\Omega }\right)$ is the position of the orthogonal spherical wave source placed below the reflecting surface.

Under the assumption that there are *M* measuring points (on the hologram plane, hereinafter defined as *H*) at field points above the reflecting plane, one can rewrite Equation (21) in a matrix form as follows.

(22) $$P^{H}=\rho c\Psi_{p,free}^{\varphi ,H}C_{j}^{\varphi }+\rho c\Psi_{p,free}^{\Omega ,H}C_{j}^{\Omega },\;=\rho c\left[\;\;\;\;\begin{array}{cc} \Psi_{p,free}^{\varphi ,H} & \Psi_{p,free}^{\Omega ,H} \end{array}\;\;\right]\;\;\left[\begin{array}{c} C_{j}^{\varphi }\\ C_{j}^{\Omega } \end{array}\right]\;=\rho c\Phi_{p,\;half}^{H}\Pi_{\Omega ,\varphi }^{H}.$$

Here, $\Psi_{p,free}^{\varphi ,H}$ and $\Psi_{p,free}^{\Omega ,H}$ is an *M × J* pressure basis function matrix connecting the pressure on *H* with the orthogonal spherical wave source point $r_{j}^{\varphi }$ and $r_{j}^{\Omega \;}$, respectively. $\Phi_{p,half}^{H}=\left[\;\begin{array}{cc} \Psi_{p,free}^{\varphi ,H} & \Psi_{p,free}^{\Omega ,H} \end{array}\;\right]$ is an *M × 2J* comprehensive pressure transfer matrix, and $\Pi_{\Omega ,\varphi }^{H}$ is a *2J × 1* column vector containing the basis function coefficients $C_{j}^{\varphi }$ and $C_{j}^{\Omega }$. The regularization of $\Pi_{\Omega ,\varphi }^{H}$ is given by

(23) $$\Pi_{\Omega ,\varphi }^{H}=\frac{1}{\rho c}{\left[{\left(\Phi_{p,half}^{H}\right)}^{H}\Phi_{p,half}^{H}+\varepsilon E\right]}^{-1}{\left(\Phi_{p,half}^{H}\right)}^{H}P^{H},$$

Any point in the half-space sound field can be reconstructed by substituting $\Pi_{\Omega ,\varphi }^{H}$ into Equation (22). The normal velocity on the surface of the vibrating object can also be reconstructed by

(24) $$V^{S}=\Phi_{v,half}^{H}\Pi_{\Omega ,\varphi }^{H},$$

where $\Phi_{v,half}^{H}$ is the $N^{S}\times 2J$ comprehensive velocity transfer matrix, like the comprehensive pressure transfer matrix $\Phi_{p,half}^{H}$. The $\Phi_{v,half}^{H}$ can be expressed as

(25) $$\Phi_{v,half}^{H}=\left[\begin{array}{cc} \Psi_{v,free}^{\varphi ,S}\; & \Psi_{v,free}^{\Omega ,S} \end{array}\;\right],$$

where $\Psi_{v,free}^{\varphi ,S}$ and $\Psi_{v,free}^{\Omega ,S}$ are expressed as

(26) $$\Psi_{v,free}^{\varphi ,S}\left(s\right)=i\frac{\partial h_{n}^{\left(1\right)}\left(kR_{j}^{\varphi S}\right)}{\partial \left(kR_{n}^{\varphi S}\right)}\sqrt{\frac{\left(2n+1\right)\left(n-m\right)!}{4\pi \left(n+m\right)!}}P_{n,m}\left(\cos\theta^{\varphi S}\right)\{\begin{array}{c} \cos\left(m\varphi^{\varphi S}\right)\\ \sin\left(m\varphi^{\varphi S}\right) \end{array},$$

(27) $$\Psi_{v,free}^{\Omega ,S}\left(s\right)=i\frac{\partial h_{n}^{\left(1\right)}\left(kR_{j}^{\Omega S}\right)}{\partial \left(kR_{n}^{\Omega S}\right)}\sqrt{\frac{\left(2n+1\right)\left(n-m\right)!}{4\pi \left(n+m\right)!}}P_{n,m}\left(\cos\theta^{\Omega S}\right)\{\begin{array}{c} \cos\left(m\varphi^{\Omega S}\right)\\ \sin\left(m\varphi^{\Omega S}\right) \end{array},$$

Here, $R_{j}^{\varphi S}$ and $R_{j}^{\Omega S}$ denote the distances between the *n*th node $r^{s}\left(x^{s},y^{s},z^{s}\right)$ on the surface *S* and the orthogonal spherical wave source points $r_{j}^{\varphi }$ and $r_{j}^{\Omega \;}$, respectively.

### 2.4. Basic Theory of E-HELS

**Figure 3:**
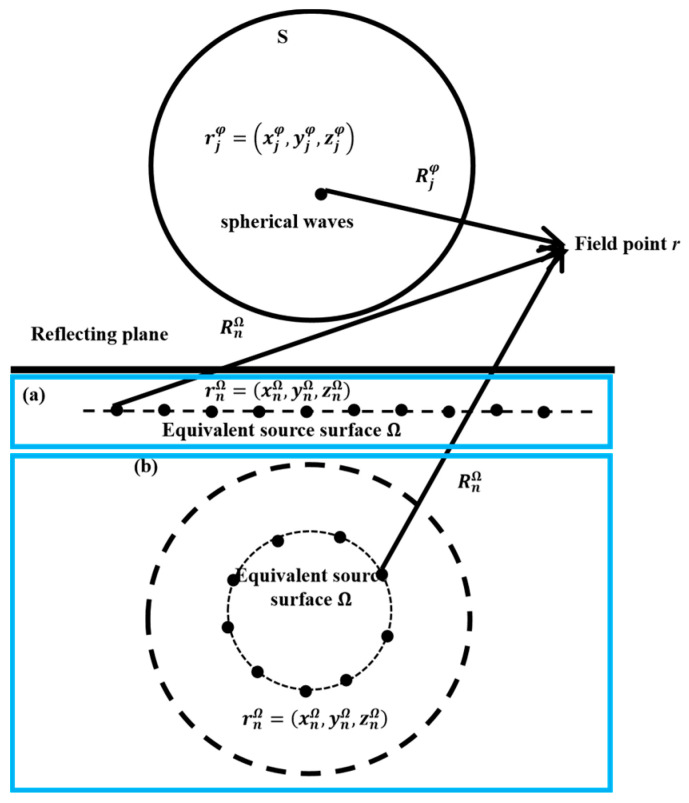
Schematic of E-HELS: (**a**) PE-HELS and (**b**) CE-HELS configurations.

In Section 2.3, spherical waves are used as an equivalent source. This section delineates the second technique, the E-HELS method. A schematic of the technique is shown in Figure 3, where the black dots below the reflecting surface represent the monopole equivalent sources, which are arranged as in (a) and (b), respectively. In the E-HELS technique, simple sources (monopoles) are used as the equivalent sources, and the reflected sound is considered to be radiated by a series of monopole sources distributed under the reflecting surface. The half-space sound field can be reconstructed by matching the hologram pressures with the direct sound of the vibrating object (approximated by a linear superposition of a series of orthogonal spherical wave sources) and with the sound reflected from the reflecting surface (approximated by a linear superposition of equivalent source strengths).

This work considers two equivalent source configurations instead of reflection, as shown in Figure 3: (a) arranged below the reflecting surface and distributed in a plane (PE-HELS) and (b) inside the image source (CE-HELS). For the PE-HELS configuration, the reflected sound is radiated by a planar sound source parallel to the reflecting surface. For the CE-HELS configuration, the reflected sound is radiated by some monopoles inside a sound source having the same size as the vibrating object, where the sound source and the vibrating object are symmetrically positioned around the reflecting surface.

In Figure 3, the pressure of the field point *r* can be approximated as

(28) $$P\left(r\right)=\rho c\sum_{j=1}^{J}\Psi_{pj,free}\left(r,r_{j}^{\varphi }\right)C_{j}+i\rho w\sum_{n=1}^{N}g_{p,free}\left(r,r_{n}^{\Omega }\right)q_{n}^{\Omega },$$

where $r_{n}^{\Omega }$ and $q_{n}^{\Omega }$ are the position and strength of the *n*th equivalent source on the virtual source plane Ω.

Similarly, one can rewrite Equation (28) in a matrix form as

(29) $$P^{H}=\rho c\Psi_{p,free}^{\varphi ,H}C_{j}^{\varphi ,H}+i\rho wG_{p,free}^{\Omega H}Q^{\Omega H}\;=\rho c\left[\;\;\;\;\begin{array}{cc} \Psi_{p,free}^{\varphi ,H} & ikG_{p,free}^{\Omega H} \end{array}\;\;\right]\;\;\left[\begin{array}{c} C_{j}^{\varphi ,H}\\ Q^{\Omega H} \end{array}\right]\;=\rho cT_{p,half}^{H}\Lambda_{\Omega ,\varphi }^{H}.$$

where $T_{p,half}^{H}=\left[\begin{array}{cc} \Psi_{p,free}^{\varphi ,H} & ikG_{p,free}^{\Omega H} \end{array}\;\right]$ is an M×(J+N) comprehensive transfer matrix, $G_{p,free}^{\Omega H}$ is the M×N pressure transfer matrix between the pressure on *H* and the equivalent sources on Ω, $Q^{\Omega H}=\left[q_{1}^{\Omega },\cdots ,q_{n}^{\Omega },\cdots ,q_{N}^{\Omega }\right]$ is a vector of the strengths of the equivalent sources on Ω, and $\Lambda_{\Omega ,\varphi }^{H}$ is the J+N vector containing the basis function coefficients $C_{j}^{\varphi ,H}$ and $Q^{\Omega H}$. The regularization of $\Lambda_{\Omega ,\varphi }^{H}$ is given by

(30) $$\Lambda_{\Omega ,\varphi }^{H}=\frac{1}{\rho c}{\left[{\left(T_{p,half}^{H}\right)}^{H}T_{p,half}^{H}+\varepsilon E\right]}^{-1}{\left(T_{p,half}^{H}\right)}^{H}P^{H}.$$

Given some field points in the half-space sound field, the pressure can be reconstructed by substituting $\Lambda_{\Omega ,\varphi }^{H}$ into Equation (29). Furthermore, the normal velocity on surface *S* can be reconstructed as

(31) $$V^{S}=T_{v,half}^{H}\Lambda_{\Omega ,\varphi }^{H},$$

where $T_{v,half}^{H}$ is an $N^{S}\times \left(J+N\right)$ comprehensive velocity transfer matrix connecting the normal velocity on *S* with the source point $r_{j}^{\varphi }$ of the orthogonal spherical wave basis function in the vibrating object and the equivalent source point $r_{n}^{\Omega }$ on Ω, respectively. It can be expressed as

(32) $$T_{v,half}^{S}=\left[\begin{array}{cc} \Psi_{v,free}^{\varphi ,S} & ikG_{v,free}^{\Omega S} \end{array}\right],$$

where $\Psi_{v,free}^{\varphi ,S}$ and $G_{v,free}^{\Omega S}$ are given by

(33) $$\Psi_{v,free}^{\varphi ,S}\left(s\right)=i\frac{\partial h_{n}^{\left(1\right)}\left(kR_{j}^{\varphi S}\right)}{\partial \left(kR_{n}^{\varphi S}\right)}\sqrt{\frac{\left(2n+1\right)\left(n-m\right)!}{4\pi \left(n+m\right)!}}P_{n,m}\left(\cos\;\theta^{\varphi S}\right)\{\begin{array}{c} \cos\left(m\varphi^{\varphi S}\right)\\ \sin\left(m\varphi^{\varphi S}\right) \end{array},$$

and $G_{v,free}^{\Omega S}$ are constituted of

(34) $$g_{v,free}\left(r^{s},r_{n}^{\Omega }\right)=\frac{\left(1-ikR_{n}^{\Omega S}\right)e^{ikR_{n}^{\Omega S}}}{4\pi {\left(R_{n}^{\Omega S}\right)}^{2}}\cos\left(\varphi_{n}^{\Omega S}\right),$$

with

(35) $$\cos\left(\varphi_{n}^{\Omega S}\right)=\frac{\left(r^{s}-r_{n}^{\Omega }\right)\cdot n^{S}}{\left|r^{s}-r_{n}^{\Omega }\right|}.$$

Here, $R_{n}^{\Omega S}$ is the distance between the *n*th node $r^{s}$ on surface *S* and $r_{n}^{\Omega }$ on Ω.

## 3. Numerical Simulation

### 3.1. Simulation Parameter Setting

**Figure 4:**
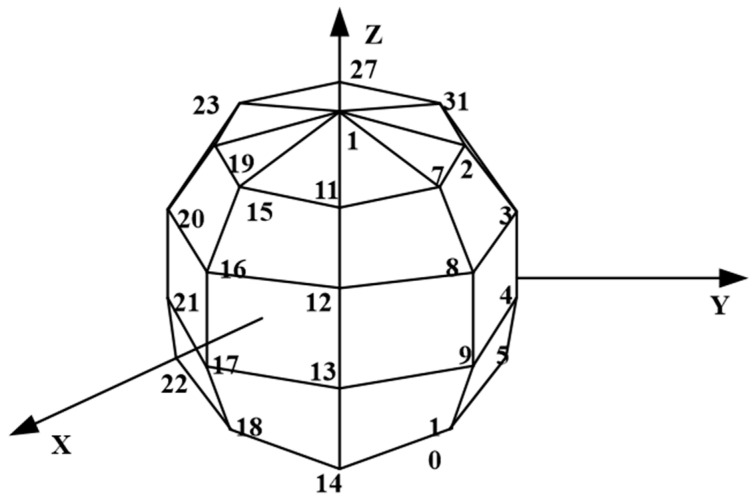
Node distribution on the surface of the vibrating ball.

A vibrating ball placed above an infinite reflector is selected as the simulation object [16] to examine the feasibility of H-HELS and E-HELS. The vibrating ball surface is uniformly divided into 34 nodes, as shown in Figure 4. The discrete intervals of the azimuth and polar angles are π/4cc and π/5.

In this simulation, it is assumed that the reflecting surface is the Delany and Bazley type [17], which is infinitely thick and reacts locally, with a surface impedance *Z* defined as

(36) $$Z=\rho c\left[1+9.08{\left(\frac{f}{\sigma_{0}}\right)}^{-0.75}+i\times 11.9{\left(\frac{f}{\sigma_{0}}\right)}^{-0.73}\right],$$

where $\sigma_{0}$ denotes the flow resistance, defined in cgs.

**Figure 5:**
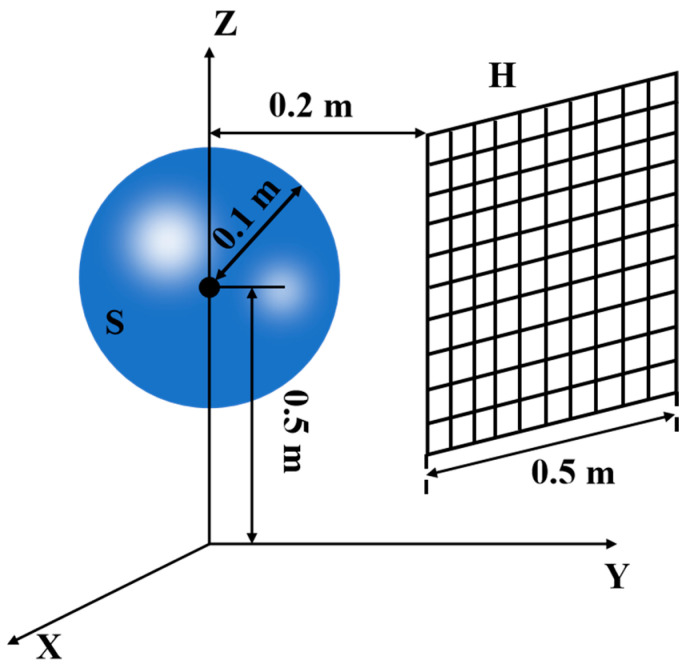
Position of the spherical source and the hologram plane *H*.

The spherical sound source is shown in Figure 5. The sphere’s center is 0.5 m above the reflecting surface, and the hologram plane *H* is perpendicular to the reflecting surface and parallel to the coordinate plane *xOz*. The center point of *H* had the same horizontal position as that of the vibrating ball, and the distance between the two center points is 0.2 m. The field points are distributed from −0.25 m to 0.25 m in the *x*-direction and from 0.25 m to 0.75 m in the *z*-direction, with sampling intervals of 0.05 m in both directions, totaling 121 measurement points.

To test the feasibility of the proposed method, the normal velocity on the surface of the vibrating ball is reconstructed. The source points of orthogonal spherical waves are positioned at the sphere’s center. For H-HELS, as mentioned in Section 2.3, an orthogonal spherical wave source is used as the equivalent source, and it is placed below the reflecting surface at *z* = −0.5 m. This wave source and the spherical surface of the vibrating ball are placed symmetrically about the reflecting surface. For E-HELS, as mentioned in Section 2.4, the monopoles are used as the equivalent source, and instead of the reflected sound, the equivalent sources distributed in the virtual source plane Ω below the reflecting surface are considered. For the PE-HELS, the equivalent source surface Ω is located at *z* = −0.001 m with an interval of 0.05 m in both directions, from −0.25 m to 0.25 m in the *x*-direction and from 0.1 m to 0.2 m in the *y*-direction. For the CE-HELS, the equivalent sources are located on the plane Ω with a radius of 0.02 m and are less than 0.4 times the radius of the vibrating sphere [18]. The normal velocity on the surface is given by

(37) $$v^{s}=v_{0}\frac{z^{s}-z_{o}}{r_{o}},$$

where $v_{0}$ is the uniform radial vibration velocity, $r_{o}$ is the radius of the vibrating sphere, and $z^{s}$ and $z_{o}$ are the *z* coordinates of the source node and source sphere center, respectively. In the simulation, the following values are set: $v_{0}=1\;m/s$, $r_{o}=0.1\;m$, and $z_{o}=0.5\;m$. The pressures on the hologram plane *H* are calculated using the boundary element method (BEM)-based half-space acoustic radiation calculation method [19] and used as the input for reconstructing the normal velocity of the vibrating ball surface. Besides, the surface impedance of the reflecting surface is set to 35 cgs units. The signal-to-noise ratio of 30 dB is added to the hologram data in the simulation to simulate the actual environment.

### 3.2. Normal Velocity Reconstruction for Vibrating Sphere

**Figure 6:**
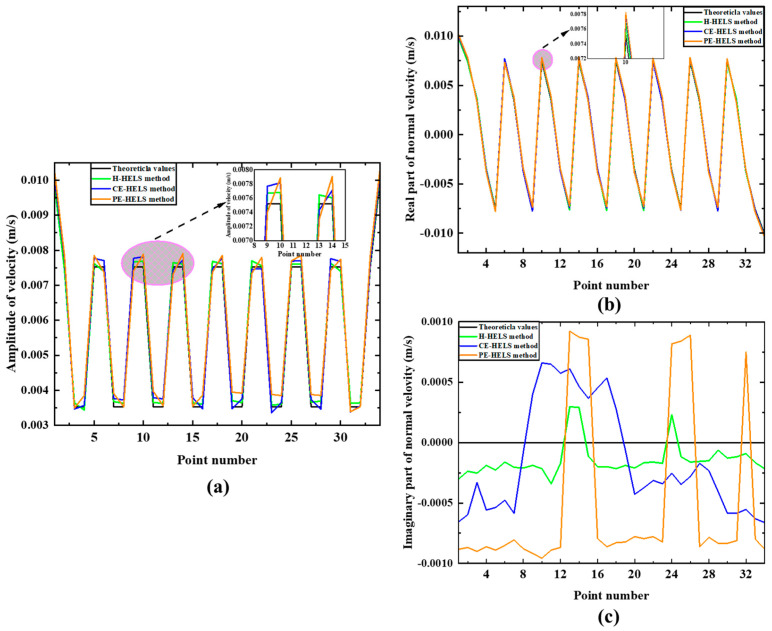
A comparison of the reconstruction velocity for the three methods: (**a**) Amplitude of the velocity, (**b**) real part, and (**c**) imaginary part.

Figure 6 shows the reconstruction results and corresponding theoretical values at 500 Hz for E-HELS and H-HELS methods. One can find that the reconstruction results of the velocity amplitude for the three methods are close to the theoretical values. Among them, the reconstruction results of the real part better agree with the theoretical values. The H-HELS method is the best among the imaginary parts of the reconstruction results, while the reconstruction results of CE-HELS are slightly worse than those of H-HELS, and PE-HELS is the worst. Overall, the reconstruction results agree with theoretical values, showing the validity of E-HELS and H-HELS for reconstructing a half-space sound field. Notably, under the least squares error control principle, the obtained solution always accurately corresponds to the measured sound pressure. Therefore, theoretically, if the measurement values are free from errors, the reconstruction accuracy of the sound pressure at the measurement points will increase as the number of expansion terms *J* increases, converging to the actual values as *J* approaches infinity. However, due to the inevitable presence of measurement errors in the measured sound pressure, there typically exists an optimal number of expansion terms, denoted as $J_{op}$, for a given set of sound field measurement data. There are two common methods for calculating *J*: the iterative method [20] and the constrained minimization method [21]. In this paper, the iterative method is used because it is more commonly used. In this simulation, the number of terms in the spherical wave expansion $J_{op}$ is set to 28, and the regularization parameter ε is 0.026.

**Figure 7:**
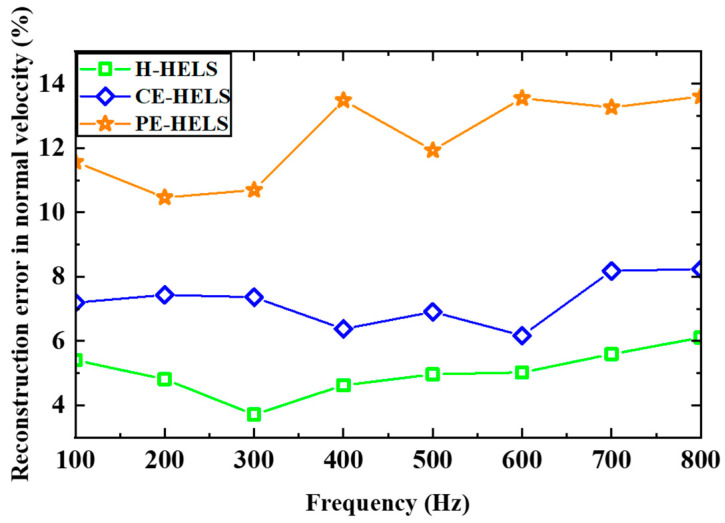
A comparison of the reconstruction velocity error versus the frequency for the three methods.

The reconstruction error for the normal surface velocity with frequency is shown in Figure 7. The reconstruction error is defined as

(38) $$E_{v}=\frac{{||V_{recon}^{s}-V_{theo}^{s}||}_{2}}{{||V_{theo}^{s}||}_{2}}\times 100,$$

where $V_{recon}^{s}$ denotes the reconstructed normal velocities on the surface of the source obtained using the three methods (H-HELS, PE-HELS, and CE-HELS), and $V_{theo}^{s}$ denotes the theoretical normal velocities obtained using the BEM-based half-space acoustic radiation calculation method. Again, the results show the effectiveness of the H-HELS and E-HELS methods. One can find from Figure 7 that H-HELS showed the highest reconstruction accuracy, with the reconstruction accuracy of CE-HELS being slightly lower and that of PE-HELS being the lowest. The reason for the reconstruction accuracy being the lowest for PE-HELS is that the reconstruction accuracy is more sensitive to the position and size of the virtual plane Ω, which is harder to determine than the other two methods. However, for CE-HELS, the position and size of the virtual plane Ω can be easily determined since it is concentric with the vibrating object. Regarding this question, it will be further discussed in Section 3.3.

**Figure 8:**
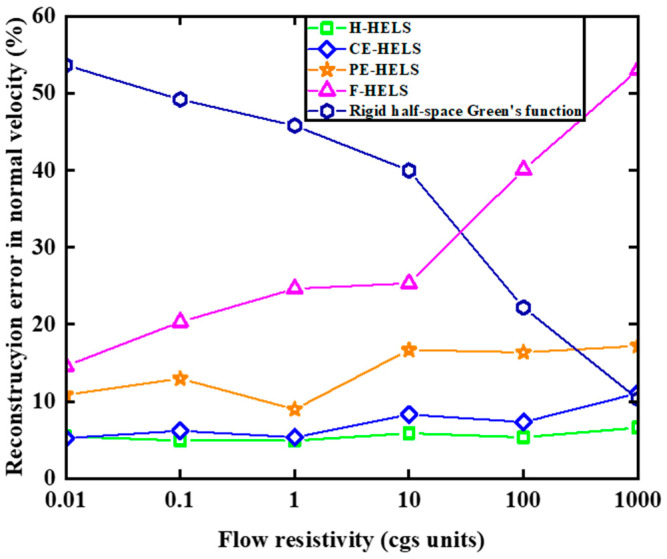
Plots of the reconstruction error in the normal relative to the spherical source surface versus the flow resistivity of the reflecting plane at 500 Hz for five methods.

If the impedance of the reflective surface is unknown, the free-space-based HELS method (F-HELS) cannot be used; however, H-HELS and E-HELS can be used. If the reflective surface is assumed to be completely rigid or absorptive, the surface normal velocity of the vibrating sphere can be reconstructed using the rigid half-space Green’s function method [22] or the F-HELS method. Figure 8 shows the surface normal velocity reconstruction error relative to the flow resistivity at 500 Hz for the vibrating ball. Apparently, the rigid half-space Green’s function and the F-HELS methods are very sensitive to the flow resistance. For a small flow resistance, the reconstruction error is large for the rigid half-space Green’s function method, and for a large flow resistance, the reconstruction error is large for the F-HELS method. In summary, these two methods cannot perform well in the entire range of flow resistance; therefore, neither method is applicable when the flow resistance of the reflective surface is unknown. However, H-HELS and E-HELS are unaffected by changes in the flow resistance of the reflecting surface, and the reconstruction accuracy is always close to that of the BEM-based half-space acoustic radiation calculation method.

### 3.3. PE-HELS Equivalent Source Configuration Analysis

As mentioned in Section 3.2, the reconstruction accuracy of PE-HELS is not high compared to CE-HESL when reconstructing the half-space sound field. The use of an improper configuration of the equivalent source on the virtual source surface Ω may result in an error. This section analyzes the effect of the equivalent source configuration of PE-HELS on the reconstruction accuracy. A knowledge of the effect can help improve PE-HELS.

**Figure 9:**
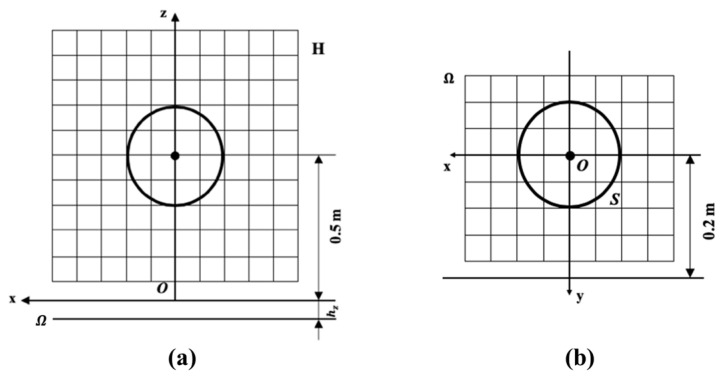
Position and dimensions of the virtual surface *Ω* and the distribution interval between two equivalent sources on *Ω* for the PE-HELS configuration: (**a**) front view and (**b**) top view.

Figure 9 shows that when the PE-HELS equivalent source configuration is used, the distance to which sound is reflected by the surface $\Omega \;\left(h_{z}\right)$ in the *z* direction, the dimensions of the surface in the *x* and *y* directions, and the equivalent source distribution interval on the surface can be determined. The variables $x_{1}$ and $x_{2}$ represent the dimension of the surface in the *x* direction,$\;y_{1}$ and $y_{2}$ represent the dimension in the *y* direction, and $d_{x}$ and $d_{y}$ represent the equivalent source distribution intervals in the *x* and *y* directions. For the simulation parameters in Section 3.1, Table 1 provides 23 sets of values for the parameter set $\left(x_{1},x_{2},y_{1},y_{2},d_{x},d_{y}\right)$.

**Figure 10:**
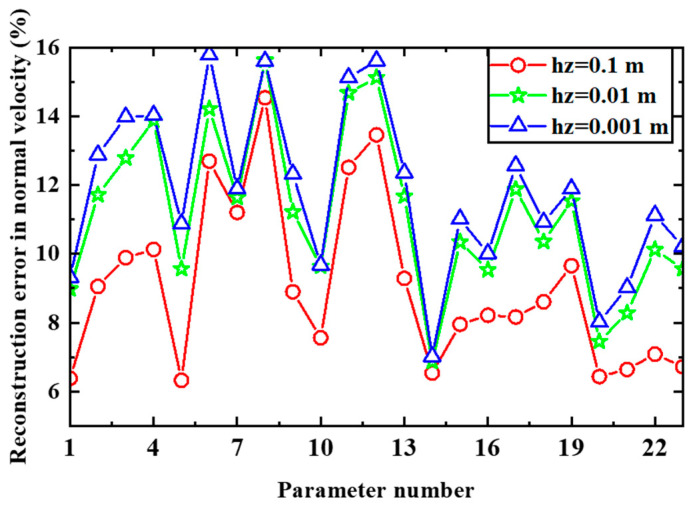
Reconstruction error in the normal velocity of the oscillating sphere surface for different values of the parameters ($h_{z},x_{1},x_{2},y_{1},y_{2},d_{x},d_{y}$).

The distance $h_{z}$ is set to 0.1, 0.01, and 0.001 m. Figure 10 shows the reconstruction error in the normal velocity of the surface when the 23 sets of parameter values in Table 1 are used. A comparison of the three curves shows that the velocity reconstruction error is small when $h_{z}$ = 0.1 m, and the distribution trend of the three curves is consistent. It indicates that the influence of the parameters $\left(x_{1},\;x_{2},\;y_{1},\;y_{2},\;d_{x},\;d_{y}\right)$ on the reconstruction error has little relation with $h_{z}$.

An analysis of the effect of the distribution interval of equivalent sources on the reconstruction error based on Table 1 and Figure 10 indicates that for the achievement of higher reconstruction accuracy, the distribution interval of the equivalent sources should be smaller when the size of the surface Ω is smaller; examples are parameter set 14 (compared to parameter set 13) and parameter set 1 (compared to parameter sets 2, 3, and 4). Conversely, when the size of the surface Ω is relatively large, the distribution interval of the equivalent sources should be large; examples are parameter set 5 (compared to parameter set 6), parameter set 7 (compared to parameter set 8), and parameter sets 9 and 10 (compared to parameter sets 11 and 12). The above parameter sets with large reconstruction errors (parameter sets 2, 3, 4, 6, 8, 11, 12, and 17) are excluded for analyzing the effect of the size of the surface Ω on the reconstruction error. It is observed that the size of the surface Ω is not too large even if the distribution interval of the equivalent sources is relatively large (e.g., parameter sets 7, 15, and 16). However, the surface Ω is considerably larger than the projection of the vibrating ball on the reflecting surface and includes the area behind the vibrating sphere. The size of the surface Ω should also not be too small, and it can be increased by making the equivalent source configuration denser; for example, the errors in parameter sets 17, 18, and 19 are relatively large, while the errors in parameter set 1 are small. Furthermore, the errors in parameter sets 20 and 21 are relatively small; for these parameter sets, the size of the surface Ω is equal to the projection of the vibrating ball on the reflecting plane. The errors in parameter sets 5, 14, 22, and 23 are relatively small, and the corresponding surface Ω covers the area between the vibrating ball and the hologram plane. This area is the main area where reflection occurs.

According to the analysis, to obtain higher reconstruction accuracy, the size of the surface Ω and the distribution interval of the equivalent sources should be appropriately set. The surface Ω just covers the gap between the vibrating objects and the hologram plane. In addition, if the surface Ω just covers the projection of the vibrating objects on the reflecting plane, higher reconstruction accuracy can be obtained, providing the distribution interval of the equivalent source is appropriate. Related research can also be found in [23].

## 4. Experiment

**Figure 11:**
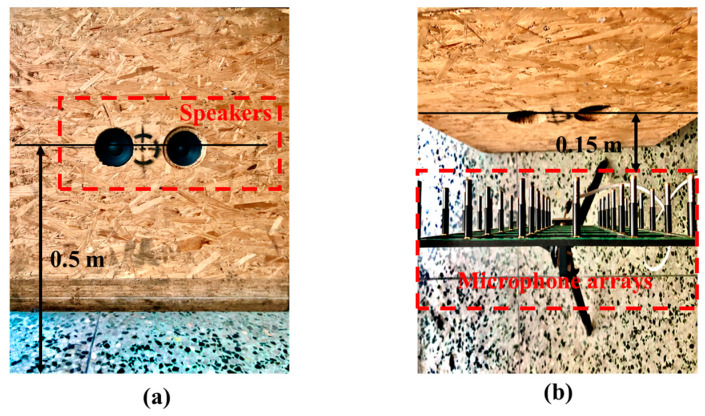
Experimental setup on the marble floor: (**a**) view of the front surface and (**b**) view of the top surface.

**Figure 12:**
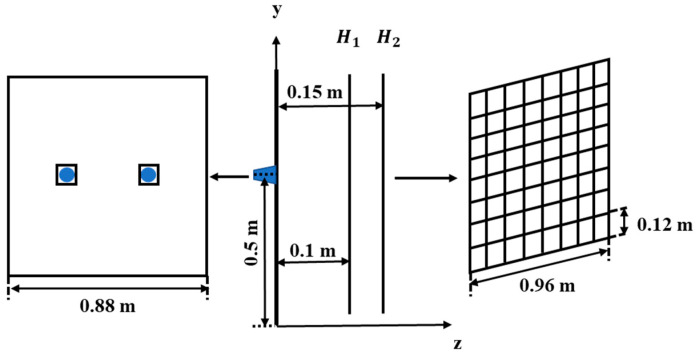
Position of the speakers and the measurement planes $H_{1}$, $H_{2}$.

**Figure 13:**
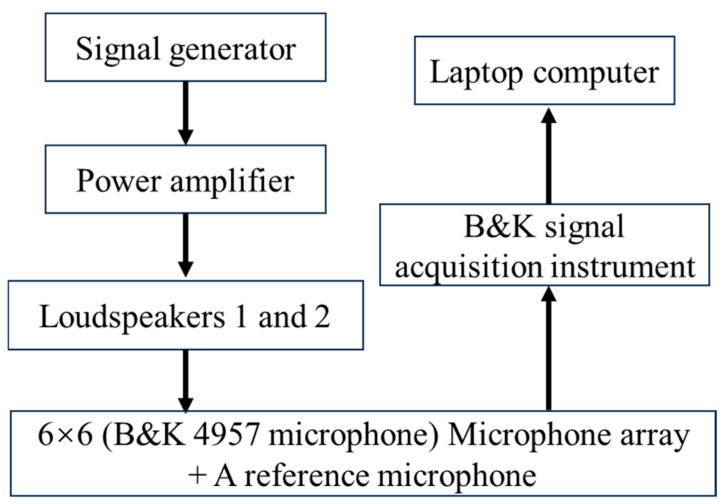
Schematic diagram of the signal acquisition system.

The experiment was conducted in an empty room. The reflecting surface was a marble floor of which the reflecting material is unknown. Two speakers were embedded in a wooden box placed on the floor. The speakers were positioned at 0.5 m above the reflecting plane, and the box dimensions were 0.88 m × 0.88 m × 0.82 m, as shown in Figure 11. The settings of the speakers (blue marker) and two measurement planes are shown in Figure 12. The canter position coordinates of the two speakers were (−0.11 m, 0.5 m, 0 m) and (0.11 m, 0.5 m, 0 m), respectively. The measurement planes $H_{1}$ and $H_{2}$ were located 0.1 and 0.15 m from the source surface, respectively. A 36-channel uniform array (B&K Model 4957) was placed 0.15 m in front of the two speakers, and the data were collected by the signal analyzer (B&K Pulse). Before and during the experiments, the phases of all microphone arrays were calibrated using a phase calibrator (B&K type 4231) and an anchor microphone. During the experiment, the speakers produced pure tones of 110 Hz and 260 Hz. The sampling frequency was 16.384 kHz, and the sampling time was 5 s. Nine separate measurements were conducted in front of the loudspeakers; each non-overlapping measurement covered the wooden box, and the dimensions of the measurement plane were 0.96 m × 0.96 m. Overall, 81 uniformly distributed data were obtained from different locations, with an interval of 0.12 m between data points in the *x* and *z* directions. Then, the transfer function between each microphone and the input signal to the loudspeakers using the averaged periodogram method [24] was obtained using the measured data. The method allowed for sufficient simultaneous measurement data to be constructed by multiplying all transfer functions by the same input signal. Figure 13 depicts this signal-acquisition process.

**Figure 14:**
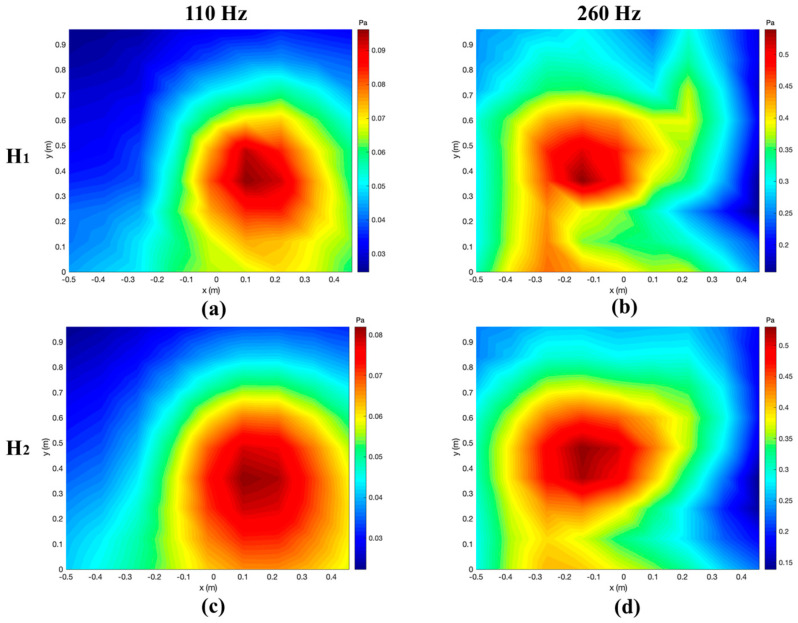
The amplitudes of the measured pressure on the plane $H_{1}$ at (**a**) 110 Hz and (**b**) 260 Hz and on the plane $H_{2}$ at (**c**) 110 Hz and (**d**) 260 Hz.

When the H-HELS method is used, the source points of the orthogonal spherical wave that replaced the direct sound radiated by the speakers are at (−0.11 m, 0.5 m, 0 m) and (0.11 m, 0.5 m, 0 m), respectively, and the source points of the orthogonal spherical wave that replaced the reflected sound are at (−0.11 m, −0.5 m, 0 m) and (0.11 m, −0.5 m, 0 m) below the reflecting surface, respectively. For E-HELS, a virtual source plane Ω is required. When the PE-HELS configuration is applied, the equivalent source plane Ω is at *y* = −0.001. Moreover, the equivalent sources are from −0.45 m to 0.45 m in the *x*-direction and from 0 m to −0.16 m in the *z*-direction, with an interval of 0.12 m in both directions. When the CE-HELS equivalent source configuration is adopted, the virtual source surface Ω is in the virtual speaker. The actual speakers and the virtual speaker are symmetric about the reflecting surface. The radius of the virtual source surface Ω is 0.4 times that of the virtual speaker. Figure 14 shows the pressures measured on two hologram planes ($H_{1}$ and $H_{2}$) at 110 and 260 Hz. One can find the speaker’s direct and reflected sound of the reflecting surface superimposed on the hologram planes.

**Figure 15:**
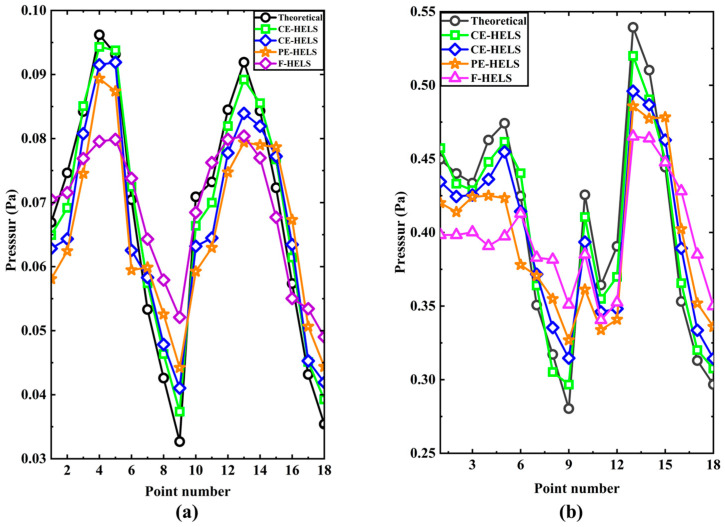
The amplitudes of the theoretical and reconstructed pressure at 16 points in the middle two columns of the reconstruction plane: (**a**) 110 Hz (x = 0.1 m, 0.2 m); (**b**) 260 Hz.

The pressures on $H_{1}$ are reconstructed using the measured pressures on $H_{2}$. The reconstructed pressures obtained by using the four methods (H-HELS, CE-HELS, PE-HELS, F-HELS) at 16 points in the middle two columns of the reconstruction plane $H_{1}$ at 110 Hz (*x* = 0.1 m, 0.2 m) and 260 Hz (*x* = −0.1 m, −0.2 m) are compared with the theoretical pressures, respectively, shown in Figure 15. One can confirm that the reconstruction result of the H-HELS method matches the measured values best, and the reconstruction using the CE-HELS also yields good results. However, the PE-HELS method is slightly worse than the CE-HELS method, although they both use equivalent sources instead of reflected sound ones. For the PE-HELS configuration, the position and size of the virtual plane Ω are difficult to determine for the best status, which results in poor results. The reconstruction results of the F-HELS method match the measured values the worst because F-HELS does not consider reflected sound, and the results are far different in some places where reflections are concentrated. In addition, the number of terms in the spherical wave expansion $J_{op}$ is set to 22 in this experiment, and the regularization parameter ε is 0.0037.

**Figure 16:**
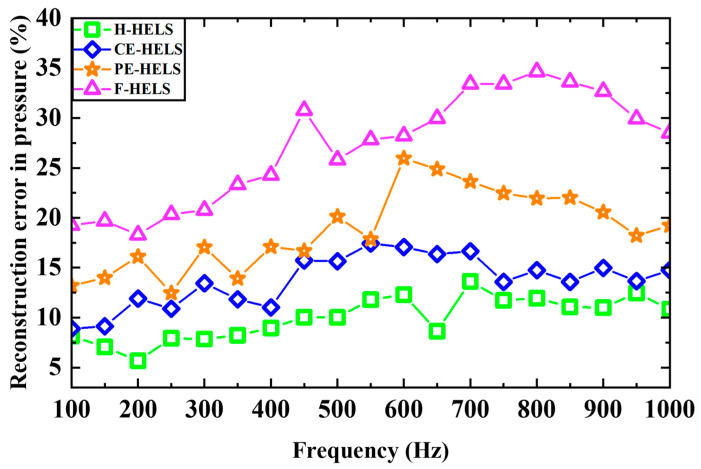
A comparison of the error of the reconstructed pressure versus the frequency using the four methods.

The reconstruction errors of the four methods on $H_{1}$ versus frequency are shown in Figure 16. The reconstruction errors are calculated using Equation (38) (the velocities replaced with the pressure). One can find that the H-HELS provides the best reconstruction results compared to the CE-HELS, PE-HELS, and F-HELS methods. The CE-HELS configuration method yields a slightly worse result than the H-HELS method, but the difference is acceptable. In contrast, the F-HELS method shows the worst performance due to the approximate rigidity of the marble floor as a reflecting surface.

## 5. Conclusions

In this work, the H-HELS and E-HELS methods are developed to reconstruct the half-space sound field above the reflecting surface when the surface impedance is uncertain. These methods are developed by introducing the concept of equivalent source in HELS-based NAH. In H-HELS, spherical waves are used as the equivalent source, and the sound reflected from the reflecting surface is regarded as a linear superposition of orthogonal spherical wave functions of different orders located below the reflecting surface. By contrast, in E-HELS, some monopoles are used as equivalent sources, and the sound reflected from the reflecting surface is regarded as a series of simple source radiations distributed under the reflecting surface. The validity and accuracy of both methods are verified through a numerical simulation of the vibrating sphere and an experiment on the marble ground. One can find that the H-HELS and E-HELS techniques successfully reconstruct the sound field over a reflecting surface with unknown surface impedance in the half-space sound field compared to the rigid half-space Green’s function and F-HELS methods. In contrast, the reconstruction results of the H-HELS method are better than the E-HELS method. With a little more error, one can consider the C-HELS method acceptable. Please note that when applying the H-HELS method, the choice of expansion coefficients particularly impacts the reconstruction accuracy.

As a solution to the problem of configuring the equivalent source in E-HELS, two configuration schemes are presented: in one configuration, PE-HELS, the equivalent sources are arranged below the reflecting surface and distributed in a plane, while in the other configuration, CE-HELS, the equivalent sources are arranged in the image source and are concentric with the image source. The simulation and experiment show that good reconstruction accuracy can be obtained using the CE-HELS equivalent source configuration. However, for the PE-HELS method, high accuracy cannot always be obtained since both the size of the virtual source plane and the backward distance of the relative reflecting surface greatly influence the reconstruction accuracy.

## Figures and Tables

**Table 1:** Parameter sets used in the simulation for the PE-HELS configuration.

Parameters	$x_{1},x_{2}\left(m\right)$	$y_{1},y_{2}\left(m\right)$	$d_{x}\left(m\right)$	$d_{y}\left(m\right)$
1	(−0.25, 0.25)	(−0.05, 0.05)	0.05	0.01
2	(−0.25, 0.25)	(−0.05, 0.05)	0.05	0.02
3	(−0.25, 0.25)	(−0.05, 0.05)	0.05	0.03
4	(−0.25, 0.25)	(−0.05, 0.05)	0.05	0.04
5	(−0.25, 0.25)	(−0.1, 0.25)	0.05	0.05
6	(−0.25, 0.25)	(−0.1, 0.25)	0.05	0.02
7	(−0.25, 0.25)	(−0.25, 0.25)	0.05	0.05
8	(−0.25, 0.25)	(−0.25, 0.25)	0.05	0.02
9	(−0.25, 0.25)	(−0.1, 0.1)	0.05	0.05
10	(−0.25, 0.25)	(−0.1, 0.1)	0.05	0.02
11	(−0.25, 0.25)	(−0.1, 0.1)	0.05	0.01
12	(−0.25, 0.25)	(−0.1, 0.1)	0.05	0.005
13	(−0.25, 0.25)	(0.1, 0.2)	0.05	0.05
14	(−0.25, 0.25)	(0.1, 0.2)	0.05	0.02
15	(−0.2, 0.2)	(−0.2, 0.2)	0.05	0.05
16	(−0.2, 0.2)	(−0.2, 0.2)	0.025	0.025
17	(−0.05, 0.05)	(−0.05, 0.05)	0.05	0.05
18	(−0.05, 0.05)	(−0.05, 0.05)	0.02	0.02
19	(−0.25, 0.25)	(−0.05, 0.05)	0.05	0.02
20	(−0.1, 0.1)	(−0.1, 0.1)	0.05	0.05
21	(−0.1, 0.1)	(−0.1, 0.1)	0.02	0.02
22	(−0.25, 0.25)	(0, 0.2)	0.05	0.05
23	(−0.25, 0.25)	(0, 0.2)	0.05	0.02

## Data Availability

The raw data supporting the conclusions of this article will be made available by the authors on request.

## References

[1] Wang Z., Wu S.F. (1997). Helmholtz equation-least-squares method for reconstructing the acoustic pressure field. J. Acoust. Soc. Am..

[2] Jiang L.X., Xiao Y.H., Zou G.P. (2019). Data extension near-field acoustic holography based on improved regularization method for resolution enhancement. Appl. Acoust..

[3] Maynard J.D., Williams E.G., Lee Y. (1985). Near-field acoustic holography: I. Theory of generalized holography and the development of NAH. J. Acoust. Soc. Am..

[4] Williams E.G. (1999). Fourier Acoustics: Sound Radiation and Near-Field Acoustical Holography.

[5] Huang L.S., Song S.Y., Xu Z.M., Zhang Z.F., He Y.S. (2020). Robust Acoustic Imaging Based on Bregman Iteration and Fast Iterative Shrinkage-Thresholding Algorithm. Sensors.

[6] Hald J. (2009). Basic theory and properties of statistically optimized near-field acoustical holography. J. Acoust. Soc. Am..

[7] Jeon I.Y., Ih J.G. (2005). On the holographic reconstruction of vibroacoustic fields using equivalent sources and inverse boundary element method. J. Acoust. Soc. Am..

[8] Zan M., Xu Z., Huang L., Zhang Z. (2020). A sound source identification algorithm based on bayesian compressive sensing and equivalent source method. Sensors.

[9] Bi C.X., Jing W.Q., Zhang Y.B., Liu W.L. (2017). Reconstruction of the sound field above a reflecting plane using the equivalent source method. J. Sound Vib..

[10] Zhao X., Wu S.F. (2005). Reconstruction of vibroacoustic fields in half-space by using hybrid near-field acoustical holography. J. Acoust. Soc. Am..

[11] Langrenne C., Melon M., Garcia A. (2007). Boundary element method for the acoustic characterization of a machine in bounded noisy environment. J. Acoust. Soc. Am..

[12] Fernandez-Grande E., Jacobsen F. (2011). Sound field separation with a double layer velocity transducer array. J. Acoust. Soc. Am..

[13] Jacobsen F., Jaud V. (2007). Statistically optimized near field acoustic holography using an array of pressure-velocity probe. J. Acoust. Soc. Am..

[14] Pan S.W., Jiang W.K., Zhang H.B., Xiang S. (2014). Modeling transient sound propagation over an absorbing plane by a half-space interpolated time-domain equivalent source method. J. Acoust. Soc. Am..

[15] Ochmann M. (2004). The complex equivalent source method for sound propagation over an impedance plane. J. Acoust. Soc. Am..

[16] Bai M.R., Ih J.G., Benesty. J. (2013). Acoustic Array Systems: Theory, Implementation, and Application.

[17] Delany M.E., Bazley E.N. (1970). Acoustical properties of fibrous absorbent materials. Appl. Acoust..

[18] Koopmann G.H., Song L., Fahnline J.B. (1989). A method for computing acoustic fields based on the principle of wave superposition. J. Acoust. Soc. Am..

[19] Marburg S., Nolte B., Bernhard R., Wang S. (2008). Computational Acoustics of Noise Propagation in Fluids: Finite and Boundary Element Methods.

[20] Wu S.F. (2000). On reconstruction of acoustic pressure fields using the Helmholtz equation least squares method. J. Acoust. Soc. Am..

[21] Lim B.D., Wu S.F. (2000). Determination of the optimal number of expansion terms in the HELS method. J. Acoust. Soc. Am..

[22] Di X., Gilbert K.E. (1993). An exact Laplace transform formulation for a point source above a ground surface. J. Acoust. Soc. Am..

[23] Jing W.Q., Wu H., Nie J.Q. (2020). Optimization of equivalent source configuration for an independent-equivalent source method in half-space sound field. Shock Vib..

[24] Welch P.D. (1967). The use of fast Fourier transform for the estimation of power spectra: A method based on time averaging over short, modified periodograms. IEEE Trans. Audio Electroacoust..

